# An Ultra-Low Dose of ∆9-Tetrahydrocannabinol Alleviates Alzheimer’s Disease-Related Cognitive Impairments and Modulates TrkB Receptor Expression in a 5XFAD Mouse Model

**DOI:** 10.3390/ijms23169449

**Published:** 2022-08-21

**Authors:** Keren Nitzan, Leah Ellenbogen, Ziv Bentulila, Dekel David, Motty Franko, Emanuela P. Break, Michal Zoharetz, Alon Shamir, Yosef Sarne, Ravid Doron

**Affiliations:** 1 Department of Education and Psychology, The Open University, Ra’anana 4353701, Israel; 2 Faculty of Medicine, Technion–Israel Institute of Technology, Haifa 3200003, Israel; 3 Mazor Mental Health Center, Akko 2423314, Israel; 4 Department of Physiology and Pharmacology, Sackler Faculty of Medicine, Tel Aviv University, Tel Aviv 6997801, Israel

**Keywords:** Alzheimer’s disease, ultra-low dose of THC, 5xFAD mice model, cognition, truncated TrkB receptor, full TrkB receptor

## Abstract

Alzheimer’s disease (AD) is the most common form of dementia, but there is still no available treatment. Δ9-tetrahydrocannabinol (THC) is emerging as a promising therapeutic agent. Using THC in conventional high doses may have deleterious effects. Therefore, we propose to use an ultra-low dose of THC (ULD-THC). We previously published that a single injection of ULD-THC ameliorated cognitive functioning in several models of brain injuries as well as in naturally aging mice. Here, 5xFAD AD model mice received a single treatment of ULD-THC (0.002 mg/kg) after disease onset and were examined in two separate experiments for cognitive functions, neurotropic, and inflammatory factors in the hippocampus. We show that a single injection of ULD-THC alleviated cognitive impairments in 6- and 12-month-old 5xFAD mice. On the biochemical level, our results indicate an imbalance between the truncated TrkB receptor isoform and the full receptor, with AD mice showing a greater tendency to express the truncated receptor, and ULD-THC improved this imbalance. We also investigated the expression of three AD-related inflammatory markers and found an ameliorating effect of ULD-THC. The current research demonstrates for the first time the beneficial effects of a single ultra-low dose of THC in a mouse model of AD after disease onset.

## 1. Introduction

Alzheimer’s disease (AD) is a progressive neurodegenerative disease characterized by the presence of extracellular plaques composed of fibrillar amyloid-β (Aβ) peptides and intracellular neurofibrillary tangles containing hyperphosphorylated tau, brain atrophy, and disrupted episodic memory function [1]. At present, there are no effective treatments. While much emphasis has been given to the amyloid and tau proteins, multiple factors are involved in the causation of AD, including mitochondrial dysfunction, oxidative stress, unbalanced iron metabolism, and neuroinflammation [2,3]. Consequently, treating the end result of the disease, such as focusing on amyloid beta (Aβ) peptides or tau phosphorylation, has not yielded satisfactory results. Instead, an effective treatment will likely involve targeting multiple aspects of the disease.

Cannabinoids are emerging as promising therapeutic agents for AD [4]. ∆9-tetrahydrocannabinol (THC), which is the major psychoactive constituent of the cannabis plant, potentially affects many of the factors involved in AD pathologies, such as Aβ clearance, mitochondrial function, inflammation, and neurogenesis [5,6]. Indeed, in vitro, THC decreases Aβ concentration in a dose-dependent manner in N2a/APPswe cells [7]. In vivo, treatment with THC reduces the Aβ burden in 5XFAD/APP mice [8] and reduces neuronal loss and restores cerebral glucose metabolism in the hippocampus of a mouse model of sporadic AD [9]. Activating the receptor of THC, which is the cannabinoid receptor (CBR), results in the removal of Aβ deposits in vitro [10] and, in vivo, improves tau and amyloid pathology in a mouse model of tauopathy [11], affects the oxidative stress response in an APP/PS1 mice model of AD, and reduces cognitive deficits in mouse and rat AD models [12,13,14,15]. However, all these studies used chronic treatments, mostly with conventional (high) doses of cannabinoid agonists (1–20 mg/kg), which can have deleterious effects [16,17]. Other than the psychoactive effect, THC exposure in young adults is correlated to deficits in attention [18], short-term memory [19], and spatial memory [20]. Therefore, a major question in the field is whether an ultra-low dose of THC (ULD-THC) can effectively treat AD while sidestepping unwanted side effects.

Previously, we observed the protective effects of ULD-THC (0.002 mg/kg, 1000–10,000 lower than conventional doses) in several models of brain injuries: in a model of epilepsy (PTZ) that impairs cognitive functions [21], in three different CNS insults that caused cognitive deficits in young mice [22], and in a mouse model of neuroinflammation [23]. We also observed reduced age-related cognitive decline in old, senile mice following a single treatment with ULD-THC [24]. THC-ULD behavioral and molecular effects were evident even seven weeks after a single administration [22]. THC reaches peak concentration in the brain after approximately 30 min, with a half-life of 2 h, while the half-life in the plasma is approximately 90 min [25]. As THC is largely fat-soluble, THC and its metabolites accumulate in fat tissue and slowly leak back into the circulation; thus, complete elimination from the body takes several days [26]. More than 80 metabolites of Δ9-THC have been identified so far, some of which may have active biological properties (such as 11-OH-Δ9-THC) while others are considered non-active [27]. Thus, most probably, the long-term effect of ULD-THC is not due to a direct action of THC. Instead, the long-term effects of ULD-THC can be explained by several mechanisms, such as the persistent expression of genes that continuously regulate the production of functional proteins long after the ULD-THC has been washed out. This has been shown before in chronic administration of THC [28]. Another possible explanation is that ULD-THC induces neurogenesis [29]. These mechanisms are probably induced by the long-lasting activation of an array of functional proteins that regulate neuroplasticity within the brain. In fact, The beneficial effects of ULD-THC were accompanied by activation of the ERK/MAPK system in the frontal cortex and the hippocampus, as well as elevation of cAMP response element-binding protein (pCREB) and brain-derived neurotrophic factor (BDNF) [22]. After identifying this long-term effect of THC on protein production, we now moved on to explore its effect on gene expression.

BDNF plays an essential role in neurons’ development, survival, and maintenance. It decreases in expression in late adulthood in correlation with a decline in hippocampal volume [30], which is connected to age-related cognitive decline [31] and AD [32]. In vitro, Aβ causes dysfunction of the BDNF signaling pathway [33], and conversely, BDNF has a protective effect on the neuronal toxicity induced by Aβ1–42 [34]. BDNF exerts its effect by binding to the tropomyosin receptor kinase B (TrkB) receptor. TrkB receptor has two main isoforms in mice—the full receptor (TrkB.FL) and the truncated isoform (TrkB.T1). TrkB.T1 lacks the kinase domain required for the classical signal transduction pathway and has an opposing activity to that of TrkB.FL [35], causing neuronal cell death and inhibiting cell regeneration and repair. TrkB.FL receptors are downregulated in the AD patient’s brain [36,37,38], while the truncated isoform of TrkB (TrkB.T1), which acts as an inhibitory modulator of BDNF signaling, is upregulated in AD [39,40].

Given our previous studies demonstrating the neuroprotective effects of ULD-THC in mice and the connection of BDNF to both AD pathology and THC’s mechanism, we hypothesized that ULD-THC would have a therapeutic effect in the 5xFAD mouse model, which shows similar characteristics to human familial-AD (FAD) patients. In addition, we explored several mechanisms that might underlie these effects.

## 2. Results

### 2.1. ULD-THC Ameliorates AD-Related Cognitive Decline

ULD-THC treatment improved the cognitive performance of AD mice at both 6 months old (during the early stages of AD) and 12 months old (advanced AD).

**Figure 1:**
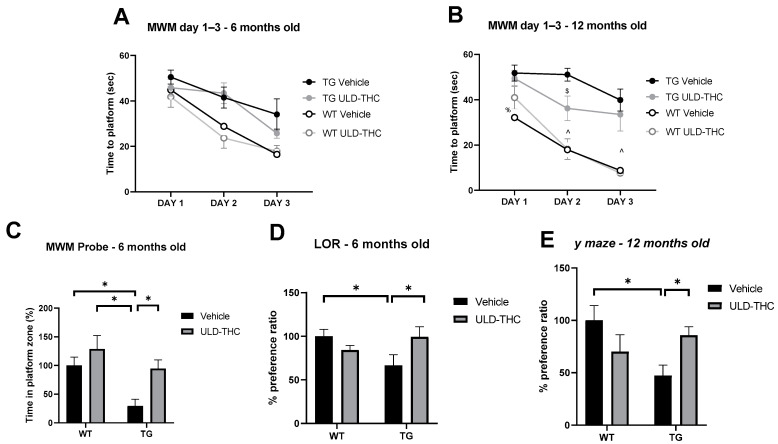
Cognitive improvement after ULD-THC. The learning of AD mice was impaired in the Morris water maze (indicative of learning ability and spatial memory) compared to WT mice, and the ULD-THC treatment alleviated this impairment at 6 months (**A**) and 12 months (**B**). On the probe phase on day 4 (**C**), 6-month-old WT mice spent significantly more time at the platform zone than AD mice, indicating the impaired long-term memory acquisition of AD mice. Importantly, ULD-THC-treated AD mice also spend more time than non-treated AD mice. In the novel location recognition test at six months (**D**), there were significant differences between the groups in long-term associative-spatial memory. Performance was impaired in AD mice compared to WT mice. Importantly, AD-treated mice exhibited a better performance compared to non-treated AD mice and spent significantly more time sniffing the object in the new location. According to the Y-maze, short-term spatial memory (**E**) was impaired in 12-month-old AD mice compared to WT mice. Importantly, treated AD mice exhibited better performance than vehicle-treated AD mice and spent significantly more time in the new arm. * indicates *p* > 0.05. ^ indicates the difference between WT groups and TG groups. %—Difference between WT control and TG groups. $—Difference between TG treated and TG control groups. Data in graphs (**C**–**E**) are presented as % from WT. *n* = 7–10. Mean ± SEM.

In the **MWM**, which measures learning ability and spatial memory, a repeated measures ANOVA test found significant differences between the groups of six-month-old mice [F (1, 3) = 5.304, *p* = 0.004]. Post-hoc analysis revealed that the vehicle-treated AD mice had slower learning than both WT groups (vehicle-treated: *p* = 0.034, ULD-THC-treated: *p* = 0.009), while the treated AD group did not differ from the WT groups (Figure 1A). Furthermore, two-way ANOVA performed on the probe phase on day 4 found a significant effect for genotype [F (1, 31) = 8.635, *p* < 0.05)] and treatment [F (1, 31) = 6.969, *p* < 0.05] at six months. Post-hoc analysis demonstrated that WT mice spent significantly more time at the platform zone than AD mice (*p* = 0.0119), indicating impaired long-term memory acquisition for the AD mice. Importantly, ULD-THC-treated AD mice spent significantly more time at the platform zone than non-treated AD mice (*p* = 0.0191) (Figure 1C).

At 12 months old in the MWM, we saw similar results. A repeated measures ANOVA test found significant differences between groups by day [(F (2.5, 60) = 1.735, *p* < 0.05]. Post-hoc testing revealed on day one a significant difference between the non-treated WT and all other groups. On day one, there was a significant difference between the non-treated WT mice and all other groups; on day three, both WT groups performed better than both 5xFAD groups (Figure 1B). Importantly, on day two, the treated AD mice outperformed the vehicle-treated AD mice, suggesting that treated AD mice learned the task faster

Similar results were obtained in the **novel location test** [F (1, 31) = 6.883], which tests long-term associative-spatial memory (Figure 1D). The performance was impaired in 6-month-old AD mice compared to WT mice (*p* = 0.02). Importantly, treated AD mice exhibited better performance than non-treated AD mice and spent significantly more time sniffing the object in the new location (*p* = 0.02).

In addition, in 12-month-old AD mice, hippocampus-dependent short-term spatial memory, tested in the **Y-maze apparatus** (Figure 1E), was impaired in the 12-month-old AD mice compared to WT mice. Importantly, treated AD mice exhibited better performance than non-treated AD mice and spent significantly more time in the new arm [F (3, 28) = 2.975 (*p* < 0.05)].

No difference was found in the first experiment (Figure S1) or the second experiment (Figure S2) between the groups in the velocity in the MWM or in the distance walked in the open field test.

### 2.2. The Effect of ULD-THC on AD-Related Imbalance of the Neurotropic Factor Receptors

**Figure 2:**
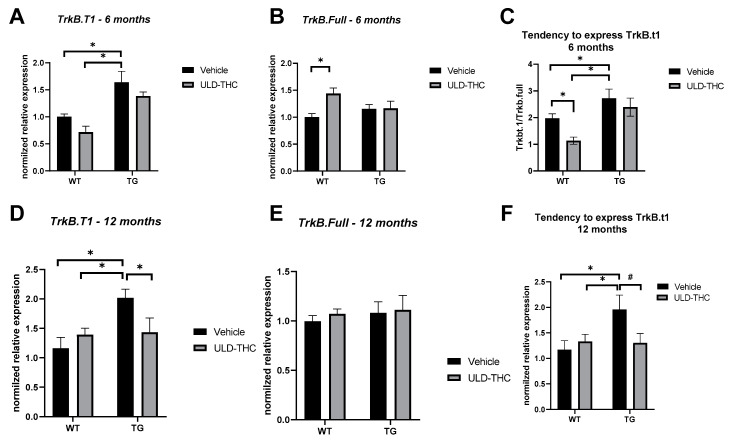
TrkB Neurotropic receptor imbalance. Six-month-old AD mice displayed higher TrkB.T1 expression in RT-PCR compared to WT mice, while no such upregulation was observed between the AD mice (**A**). There was no difference in the gene expression levels of TrkB-full receptor between AD mice and WT mice, although we did see an upregulation in the ULD-THC-treated WT mice compared to the non-treated WT mice (**B**). Non-treated AD mice had a higher ratio of expressed TrkB.T1: TrkB-full compared to WT mice, while no such tendency was observed in treated AD mice (**C**). 12-month-old AD mice displayed higher TrkB.T1 expression compared to WT mice, while no such upregulation was observed between the treated AD mice (**D**). There was no difference in the gene expression levels of TrkB-full receptor between AD mice and WT mice, although we did see an upregulation in the ULD-THC-treated WT mice compared to the non-treated WT mice (**E**). Non-treated AD mice had a higher ratio of expressed TrkB.T1: TrkB-full compared to WT mice, while no such tendency was observed in treated AD mice (**F**). * indicates *p* > 0.05. # indicates *p* = 0.08 Mean ± SEM.

Next, we set out to explore a possible molecular mechanism for the beneficial effect of ULD-THC. Given its known effect on BDNF and BDNF’s receptors, we assessed the relative expression (Delta-delta values) of the full and truncated variants of the TrkB receptor in the hippocampal tissue of WT, treated WT, AD and treated AD mice. One-way ANOVA and Tukey’s multiple comparisons test on the expression of TrkB-trunked receptor (TrkB.T1) found that the AD mice had more TrkB.T1 expression than WT mice (*p* = 0.002), while there was no such upregulation in the treated AD mice of both age groups (6 months old: F (1, 31) = 30.87, *p* < 0.001, Figure 2A). Twelve months old: [F (1, 13) = 6.104, *p* = 0.02, Figure 2D)]. There was no difference in the gene expression levels of TrkB-full receptor between AD mice and WT mice (Figure 2B,E), although we observed an upregulation in the ULD-THC-treated WT mice compared to the non-treated WT mice [F (1, 31) = 4.507, *p* = 0.01)].

We also calculated the ratio between the TrkB-full and TrkB.T1 [41] in the different groups (TrkB.T1/TrkB-full; higher score = expressing the truncated version over the full version of the receptor). At six months old (Figure 2C), there was a significant difference between the groups [F (1, 28) = 12.91, *p* = 0.0012)], with the non-treated AD mice expressing a higher ratio of TrkB.T1:TrkB-full than WT mice, while no such tendency was observed in treated AD mice (*p* < 0.05). At 12 months old (Figure 2F), there was a trend toward a difference between the groups [F (1, 13) = 3.369, *p* = 0.08)], with the non-treated AD mice expressing a higher ratio than WT mice (*p* = 0.02), and a trend towards a lower ratio in treated AD mice (*p* = 0.06).

### 2.3. The Effect of ULD-THC on AD-Related Inflammatory Markers

**Figure 3:**
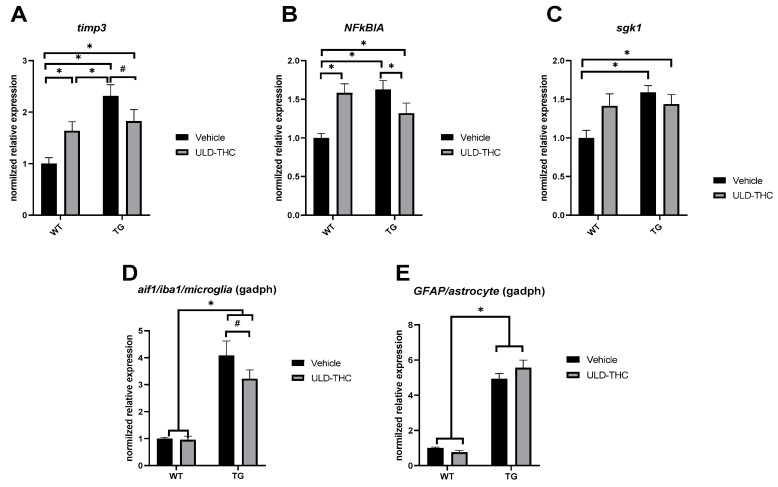
Inflammatory and glia markers of AD at six months. Relative expression in RT-PCR of Tissue Inhibitor of Metalloproteinase 3′ (TIMP-3) was elevated in non-treated AD mice compared to WT mice. Importantly, AD-treated mice had lower expression compared to non-treated mice (**A**). Relative expression of the Nfkbia gene was elevated in non-treated AD mice compared to WT mice. Importantly, AD-treated mice had lower expression compared to non-treated mice (**B**). Relative expression of Glucocorticoid activated kinase (Sgk) was higher in non-treated AD mice and in AD-treated mice compared to WT mice (**C**). The relative expression of both aif1 gene (**D**), related to microglia and GFAP gene (**E**); related astrocytes were upregulated in non-treated AD mice compared to WT mice. Notably, in the aif1 gene, AD-treated mice showed a trend toward less gliosis compared to non-treated mice. *n* = 7–10. * indicates *p* > 0.05, # indicates *p* = 0.06, Mean ± SEM.

To further elucidate the impact of ULD-THC on AD, we investigated the relative expression (Delta-delta values) of three AD-related inflammatory markers. First, we examined a tissue inhibitor of metalloproteinase 3 (TIMP-3) (Figure 3A). TIMP-3 inhibits APP cleavage, resulting in increased levels of Aβ, and is upregulated in the brains of both human AD patients and AD mice models. As expected, there was a significant difference between the groups [F (1, 31) = 4.476, *p* = 0.0425]. Non-treated AD mice exhibited highly elevated expression levels of TIMP-3 compared to WT mice (*p* < 0.001). Importantly, AD-treated mice had lower expression compared to non-treated AD mice (*p* = 0.05). Glucocorticoid activated kinase (SGK) is related to tau pathology and also participates in the signaling of BDNF. It is upregulated by a variety of hormones, including mineralocorticoids and glucocorticoids. We saw a significant difference between the groups [F (1, 32) = 5.547, *p* = 0.0248], with AD mice showing higher Sgk1 expression compared to WT mice (*p* = 0.0082) (Figure 3C). Treated mice also showed higher expression compared to WT (*p* = 0.05). The Nfkbia gene (encoding NFKB inhibitor alpha) is another inflammatory marker that is highly elevated in AD. As expected, we saw a significant difference between the groups [F (1, 31) = 17.30, *p* = 0.0002], with non-treated AD mice exhibiting highly elevated expression compared to WT mice (*p* < 0.001). Importantly, AD-treated mice had lower expression compared to non-treated AD mice (*p* = 0.05) (Figure 3B).

Gliosis is also a known AD pathology. Thus, we looked both at microglia (using the Aif1 gene that is related to IBA1, a microglia marker) and at astrocytes (using the gene for GFAP) (Figure 3D,E). Both genes were upregulated in AD mice compared to WT mice ([F (1, 30) = 76.13, *p* < 0.001]; [F (1, 31) = 175.6, *p* < 0.001], respectively), which is indicative of the expected gliosis in the AD group. Notably, for the aif1 gene, the treated AD mice showed a trend toward less gliosis compared to non-treated mice (*p* = 0.06).

## 3. Discussion

In this study, we show for the first time the protective effect of a single treatment of ULD-THC in an AD mouse model. A group of 5xFAD mice receiving a single treatment of ULD-THC (0.002 mg/kg, 3–4 orders of magnitude lower than conventional doses) after disease onset showed improved cognitive performance compared to non-treated AD mice, both in short-term and long-term spatial memory tests.

Our results coincide with recently published research. Lower doses of THC have been recently shown to be beneficial in the APP/PS1 mouse model of AD given both i.p. and intranasally [14,15]. However, the experiment used very old mice (14 months old) and importantly, they used a chronic daily THC treatment for three months. However, numerous studies have shown that high or chronic doses of THC may have deleterious effects [16,17]. For a more comprehensive review of the possible deleterious effects of THC, see our recent publication [42].

For this reason, we examined the effect of a single acute treatment. We previously observed the protective effects of ULD-THC in several models of brain injuries: in a model of epilepsy (PTZ) that impairs cognitive functions [21], in three different CNS insults that cause cognitive deficits in young mice [22], and in a mouse model of neuroinflammation, in which ULD-THC was protective against cognitive deficits [23]. A single dose of ULD-THC produced a protective effect with a wide therapeutic time window, and the drug could be effectively introduced from 1–7 days before or after the insult [23]. We also observed a reduction of age-related cognitive decline in old, senile mice following a single treatment with ULD-THC [24]. Similarly, and continuing our previous line of research, here we saw an improvement in the cognitive performance of AD-model mice treated with a single dose of ULD-THC.

This research shows the beneficial behavioral and biochemical effects of a single dose of ULD-THC in AD. ULD-THC improved the cognitive deficits after the disease onset in 5xFAD mice. However, this is just the first step in elucidating the beneficial effect of ULD-THC (also observed by other researchers [14,15]). Further investigation should explore how long this one-time treatment work, as well as explore chronic administration earlier, before the disease onset.

Our previous work showed that the neuroprotective effect of ULD-THC is accompanied by prolonged activation of signaling pathways that mediate neuronal plasticity, survival, and proliferation [42]. Its beneficial effects were accompanied by activation of the ERK/MAPK system in the frontal cortex and the hippocampus, as well as elevation of cAMP response element-binding protein (pCREB) and brain-derived neurotrophic factor (BDNF) [22].

BDNF is associated with several AD treatments. BDNF is produced by astrocytes [43] and neurons [44] and is a crucial mediator of neuronal plasticity [45] and neurogenesis [46,47]. BDNF plays an essential role in the development, survival, and maintenance of neurons and has been shown to decrease in late adulthood in correlation with a decline in hippocampal volume [30], and is also connected to age-related cognitive decline [31] and AD [32]. In vitro, Aβ causes dysfunction of the BDNF signaling pathway [33], while BDNF demonstrates a protective effect on neuronal toxicity induced by Aβ1-42 [34]. BDNF exerts its effect by binding to the TrkB receptor. 

The TrkB receptor has two main isoforms in mice—the full receptor (TrkB.FL) and the truncated isoform (TrkB.T1). TrkB.T1 lacks the kinase domain required for the classical signal transduction pathway and has an opposing activity to that of TrkB.FL [35], causing neuronal cell death and inhibiting cell regeneration and repair. TrkB.T1 is upregulated in multiple disease states such as chronic pain [48], Alzheimer’s disease [39] and Parkinson’s disease [49]. The TrkB.full/Trk.T1 ratio has been shown to have clinical importance. The TrkB.full/Trk.T1 ratio has been shown to be connected to stress-induced cognitive decline [41], and in schizophrenia patients, this ratio has a predictive value as a lower ratio in the periphery (lower expression of the full receptor and higher truncated expression) was associated with worse clinical response to an antipsychotic [50]. Our results here indicate an imbalance between the truncated receptor isoform and the full receptor, with AD mice showing a greater tendency to express the truncated receptor in the hippocampus both at 6 and 12 months. Thus, one possible explanation for our results could be that ULD-THC downregulates the TrkB.T1 isoform, correcting AD-related imbalance between the TrkB isoforms.

This research shows the beneficial biochemical effects of a single dose of ULD-THC in AD. We investigated the effect on RNA expression after our previous publication indicating that ULD-THC affects protein production [22]. However, it should be noted that gene expression and protein expression do not always correlate; thus, additional research should explore the effect of ULD-THC on TrkB protein expression and on more classical hallmarks of AD, such as Aβ accumulation.

Aside from neurotropic system imbalance, AD mice exhibited higher expression levels of both iba1- and gfap-related genes, indicating gliosis in this model, while the treated AD mice showed a trend toward less gliosis, specifically in microglia, compared to non-treated mice. During the last decade, the microglial contribution to the development and progress of AD pathology has been widely recognized and accepted. Microglia have been connected to AD-related inflammatory cytokine release and cellular death [51]. Interestingly, microglial activity is also important in regulating the neurotropic system [52]. Thus, the balancing of the TrkB receptor that we saw here might be connected to the reduction in gliosis, although the exact nature of this interaction is not yet clear. AD-treated mice also had lower inflammatory related-genes expression than non-treated AD mice. We have previously shown that the beneficial effects of ULD-THC were mediated by CB1R [23]. CBRs are involved in modulating glial activation [53]. This may explain the anti-inflammatory effect we saw in ULD THC-treated AD mice.

It should be noted that WT mice treated with ULD-THC exhibited some heightened expression of inflammatory related-genes compared to untreated WT mice. This is in accordance with the notion of the dual-effect of ULD-THC; where ULD-THC given to young and healthy mice demonstrates cognitive decline [54], whereas in aged or neurologically impaired mice, ULD-THC shows beneficial effects [23,24]. This age-dependent dual effect of low doses of THC was suggested to be related to CB1 activation, histone acetylation, as well as to the expression of BDNF and TrkB [28] (for an extensive review, see [42,55,56]).

## 4. Materials and Methods

### 4.1. Animals

We performed two separate and independent experiments using the 5xFAD mouse model (Swedish K670N, M671L, Florida I716V, London V717I, and two mutations in the human presenilin-1 gene: M146L and L286V; stock number 34840) [57]. The first experiment was performed on six-month-old female 5xFAD tg-mice (provided by Prof. Alon Monsonego, Ben-Gurion University, Israel) and their wildtype (WT) littermates (*n* = 8–10 per treatment group). The second experiment was performed on 12-month-old 5xFAD tg-mice of both sexes to ensure adequate sample sizes (provided by Prof. Nadir Arber, The Integrated Cancer Prevention Center, Tel Aviv Sourasky Medical Center, Tel Aviv, Israel), as well as their wildtype (WT) littermates (n = 8–9 per treatment group, mixed sex. See Table S1 and Figure S3 in Supplementary Materials for more information).

Mice were genotyped by PCR analysis of tail DNA to identify the tg-mice, and the non-tg littermates were used as control WT mice. Mice were kept in the vivarium at the Ein Kerem Hadassah Medical Center’s Psychobiology Lab of the Open University on a reverse 12 h light/dark cycle and provided with food and water ad libitum. All experiments were performed during the dark phase (7:00–19:00) under red light. All experimental protocols were examined and approved by the Institutional Animal Care and Use Committee.

### 4.2. Treatments

Δ9-THC (donated by Prof. Mechoulam, the Hebrew University, Jerusalem and by NIDA, USA) was dissolved from a stock solution in ethanol into a vehicle solution consisting of 1:1:18 ethanol:cremophor (Sigma-Aldrich, Rehovot, Israel): saline and was administered i.p. at a dose of 0.002 mg/kg. Mice were treated with a single i.p. injection of either ULD-THC or vehicle and tested three weeks later.

### 4.3. Behavioral Tests

**Morris Water Maze (MWM):** Learning ability and spatial memory were evaluated using MWM. This classical spatial learning assay was performed as previously described [24]. Briefly, the mouse was introduced into a round pool at different starting points and allowed 60 s to find the platform. Water was kept at the temperature of 23 degrees Celsius [58]. Each mouse swam four times per day for three consecutive days. The time and route required for the mouse to find the immersed platform was recorded. On the last day of training, velocity was measured to determine intact motor function. On day 4 of the assay, the platform was removed, and the mouse was allowed to swim for 60 s (“probe test”). The time the mice spent in the platform zone (the location of the missing platform) was recorded.

**Y-maze (YM):** The YM assay is based on the tendency of mice to explore a new environment. The maze comprises three 30 cm long and 120° apart identical arms, with each arm presenting different visual cues. The mouse was placed at the distal edge of the start arm during the first session, with one of the two remaining arms blocked and left to explore the maze for 5 min. The mouse was then returned to its home cage and reintroduced into the start arm 2 min later for 2 min, with the two remaining arms open. The total time that the mouse spent exploring each of the two arms was recorded, normalized, and used to calculate the preference index [24]. Data are presented as a percentage from the WT group.

**Object Place recognition tests:** This assay tests long-term memory and utilizes the natural tendency of mice to explore novel stimuli. They were performed as previously described [24]. Briefly, the test comprises two parts: a familiarization session and a test session. During the familiarization session, the mouse was left to explore two identical objects for 5 min. 24 h later, the mouse was introduced into the arena for the test session, in which one of the familiar objects was placed in a new location (“place recognition”). The time spent exploring each object was recorded for 5 min, and the relative time (RT) of exploration was calculated as the time spent exploring the novel object over the total exploration time for both objects. Data are presented as a percentage of the WT group.

**Open field test (OFT):** The OFT tests motor function and consists of an empty square arena (40 × 40 × 40 cm) surrounded by Perspex opaque walls. Mice were placed in the center of the arena, and their behavior was video recorded for a total of 5 min. The maze was thoroughly cleaned with ethanol and allowed to dry between subjects to eliminate odor cues. The distance traveled by the mice measured motor behavior.

All the behavioral assays were photographed using a video camera, and data were recorded and analyzed using the 13th Noldus EthoVision software.

### 4.4. RT-PCR

After the last behavioral test, mice were sacrificed, and the hippocampus tissue was removed. According to the manufacturer’s instructions, total RNA was extracted from the hippocampus using the PureLink RNA Mini Kit (Rhenium, Mod’din, Israel). SYBR Green real-time PCR primers were purchased from Agentek Israel. RT-PCR was performed with primers specific for tropomyosin receptor kinase B (TrkB) and truncated tropomyosin receptor kinase B (TrkB.T1). The tissue inhibitor of metalloproteinase 3 (Timp-3), Sgk1, nuclear factor kappa-light-chain-enhancer of activated B cells inhibitor alpha (Nfkbia), allograft inflammatory factor 1 (Aif1), glial fibrillary acidic protein (Gfap) (Agantek, Yakum, Israel) were assayed for expression using the Magnetic Induction Cycler (Mic) PCR Machine (Biomolecular Systems, Upper Coomera, Australia). Delta-delta values were calculated compared to the Gapdh gene.

### 4.5. Study Design

**Figure 4:**
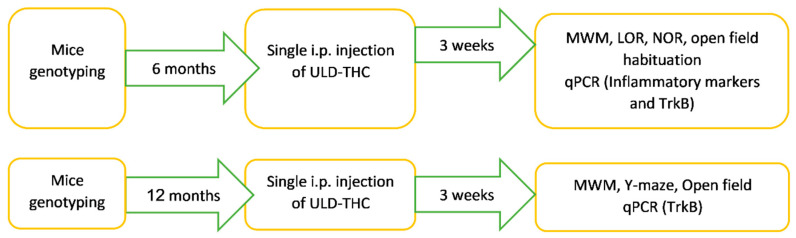
Study design. The 5xFAD tg mice model at different ages and their WT littermates were treated with a single i.p. injection of either ULD-THC or a vehicle and tested for short- and long-term spatial memory three weeks later.

To determine the treatment efficacy of ULD-THC in the 5xFAD tg mice model, 6-month-old female 5xFAD mice (experiment 1) or 12-month-old male and female 5xFAD mice (experiment 2) and their WT littermates were treated with a single i.p. injection of either ULD-THC or vehicle and tested for short and long-term spatial memory three weeks later (Figure 4). The experiments with older mice used both sexes to achieve an adequate sample size.

### 4.6. Data Analysis

All results are presented as mean ± standard error of the mean. Behavioral data were analyzed using ANOVA and further analyzed using post-hoc analysis. Extreme values (±2 standard divisions) were excluded from the statistical analysis. Statistical analysis was performed using IBM SPSS Statistics V.25. The level of significance was set at *p* < 0.05.

## 5. Conclusions

The current research demonstrates for the first time the beneficial effects of a single ultra-low dose of THC in a mouse model of AD after disease onset. It also confirms our previous reports regarding the age-depended dual effect of ULD-THC [54]. These effects are probably not a result of a direct action of THC; rather, the long-term effects of ULD-THC are most probably explained by mechanisms, such as the persistent expression of genes, that continuously regulate the production of functional proteins long after the ULD-THC has been washed out. Further studies should investigate other dosing regimens as well as other possible AD-related outcomes. As THC is a cheap, widely available substance already approved for use in other conditions, this research brings us closer to understanding its mechanisms and will possibly lead to new treatments.

## Data Availability

The datasets used and/or analyzed during the current study are available from the corresponding author on reasonable request.

## Supplementary Materials

The following supporting information can be downloaded at: https://www.mdpi.com/article/10.3390/ijms23169449/s1.

## References

[1] Goedert M., Spillantini M.G. (2006). A Century of Alzheimer’s Disease. Science.

[2] Iqbal K., Grundke-Iqbal I. (2010). Alzheimer’s Disease, a Multifactorial Disorder Seeking Multitherapies. Alzheimer’s Dement..

[3] Frenkel D. (2020). Alzheimer’s Disease: A Need for Personalized Therapeutic Approaches. Drug Dev. Res..

[4] Kendall D.A., Yudowski G.A. (2017). Cannabinoid Receptors in the Central Nervous System: Their Signaling and Roles in Disease. Front. Cell. Neurosci..

[5] Talarico G., Trebbastoni A., Bruno G., de Lena C. (2019). Modulation of the Cannabinoid System: A New Perspective for the Treatment of the Alzheimer’s Disease. Curr. Neuropharmacol..

[6] Kim S.H., Yang J.W., Kim K.H., Kim J.U., Yook T.H. (2019). A Review on Studies of Marijuana for Alzheimer’s Disease—Focusing on CBD, THC. J. Pharmacopunct..

[7] Cao C., Li Y., Liu H., Bai G., Mayl J., Lin X., Sutherland K., Nabar N., Cai J. (2014). The Potential Therapeutic Effects of THC on Alzheimer’s Disease. J. Alzheimer’s Dis..

[8] Chen R., Zhang J., Fan N., Teng Z., Wu Y., Yang H., Tang Y., Sun H., Song Y., Chen C. (2013). Δ9-THC-Caused Synaptic and Memory Impairments Are Mediated through COX-2 Signaling. Cell.

[9] Franke T., Irwin C., Beindorff N., Bouter Y., Bouter C. (2019). Effects of Tetrahydrocannabinol Treatment on Brain Metabolism and Neuron Loss in a Mouse Model of Sporadic Alzheimer’s Disease. Nuklearmedizin.

[10] Tolón R.M., Núñez E., Pazos M.R., Benito C., Castillo A.I., Martínez-Orgado J.A., Romero J. (2009). The Activation of Cannabinoid CB2 Receptors Stimulates in Situ and in Vitro Beta-Amyloid Removal by Human Macrophages. Brain Res..

[11] Casarejos M.J., Perucho J., Gomez A., Muñoz M.P., Fernandez-Estevez M., Sagredo O., Fernandez Ruiz J., Guzman M., de Yebenes J.G., Mena M.A. (2013). Natural Cannabinoids Improve Dopamine Neurotransmission and Tau and Amyloid Pathology in a Mouse Model of Tauopathy. J. Alzheimer’s Dis..

[12] Haghani M., Shabani M., Javan M., Motamedi F., Janahmadi M. (2012). CB1 Cannabinoid Receptor Activation Rescues Amyloid SS-Induced Alterations in Behaviour and Intrinsic Electrophysiological Properties of Rat Hippocampal CA1 Pyramidal Neurones. Cell. Physiol. Biochem..

[13] Wu J., Bie B., Yang H., Xu J.J., Brown D.L., Naguib M. (2013). Activation of the CB2 Receptor System Reverses Amyloid-Induced Memory Deficiency. Neurobiol. Aging.

[14] Fihurka O., Hong Y., Yan J., Brown B., Lin X., Shen N., Wang Y., Zhao H., Gordon M.N., Morgan D. (2022). The Memory Benefit to Aged APP/PS1 Mice from Long-Term Intranasal Treatment of Low-Dose THC. Int. J. Mol. Sci..

[15] Wang Y., Hong Y., Yan J., Brown B., Lin X., Zhang X., Shen N., Li M., Cai J., Gordon M. (2022). Low-Dose Delta-9-Tetrahydrocannabinol as Beneficial Treatment for Aged APP/PS1 Mice. Int. J. Mol. Sci..

[16] Cohen K., Weinstein A. (2018). The Effects of Cannabinoids on Executive Functions: Evidence from Cannabis and Synthetic Cannabinoids—A Systematic Review. Brain Sci..

[17] Ballinger M.D., Saito A., Abazyan B., Taniguchi Y., Huang C.-H., Ito K., Zhu X., Segal H., Jaaro-Peled H., Sawa A. (2015). Adolescent Cannabis Exposure Interacts with Mutant DISC1 to Produce Impaired Adult Emotional Memory. Neurobiol. Dis..

[18] Miller R.L.A., Thakur G.A., Stewart W.N., Bow J.P., Bajaj S., Makriyannis A., McLaughlin P.J. (2013). Effects of a Novel CB1 Agonist on Visual Attention in Male Rats: Role of Strategy and Expectancy in Task Accuracy. Exp. Clin. Psychopharmacol..

[19] Hampson R.E., Deadwyler S.A. (2000). Cannabinoids Reveal the Necessity of Hippocampal Neural Encoding for Short-Term Memory in Rats. J. Neurosci..

[20] Verrico C.D., Gu H., Peterson M.L., Sampson A.R., Lewis D.A. (2014). Repeated Δ9 -Tetrahydrocannabinol Exposure in Adolescent Monkeys: Persistent Effects Selective for Spatial Working Memory. Am. J. Psychiatry.

[21] Assaf F., Fishbein M., Gafni M., Keren O., Sarne Y. (2011). Pre- and Post-Conditioning Treatment with an Ultra-Low Dose of Δ9-Tetrahydrocannabinol (THC) Protects against Pentylenetetrazole (PTZ)-Induced Cognitive Damage. Behav. Brain Res..

[22] Fishbein M., Gov S., Assaf F., Gafni M., Keren O., Sarne Y. (2012). Long-Term Behavioral and Biochemical Effects of an Ultra-Low Dose of Δ9-Tetrahydrocannabinol (THC): Neuroprotection and ERK Signaling. Exp. Brain Res..

[23] Fishbein-Kaminietsky M., Gafni M., Sarne Y. (2014). Ultralow Doses of Cannabinoid Drugs Protect the Mouse Brain from Inflammation-Induced Cognitive Damage: Ultralow THC Doses Protect Against Inflammation-Induced Cognitive Damage. J. Neurosci. Res..

[24] Sarne Y., Toledano R., Rachmany L., Sasson E., Doron R. (2018). Reversal of Age-Related Cognitive Impairments in Mice by an Extremely Low Dose of Tetrahydrocannabinol. Neurobiol. Aging.

[25] Torrens A., Vozella V., Huff H., McNeil B., Ahmed F., Ghidini A., Mahler S.V., Huestis M.A., Das A., Piomelli D. (2020). Comparative Pharmacokinetics of Δ9-Tetrahydrocannabinol in Adolescent and Adult Male Mice. J. Pharmacol. Exp. Ther..

[26] Agurell S., Halldin M., Lindgren J.E., Ohlsson A., Widman M., Gillespie H., Hollister L. (1986). Pharmacokinetics and Metabolism of Delta 1-Tetrahydrocannabinol and Other Cannabinoids with Emphasis on Man. Pharmacol. Rev..

[27] Dinis-Oliveira R.J. (2016). Metabolomics of Δ9-Tetrahydrocannabinol: Implications in Toxicity. Drug Metab. Rev..

[28] Bilkei-Gorzo A., Albayram O., Draffehn A., Michel K., Piyanova A., Oppenheimer H., Dvir-Ginzberg M., Rácz I., Ulas T., Imbeault S. (2017). A Chronic Low Dose of Δ9-Tetrahydrocannabinol (THC) Restores Cognitive Function in Old Mice. Nat. Med..

[29] Marchalant Y., Brothers H.M., Wenk G.L. (2009). Cannabinoid Agonist WIN-55,212-2 Partially Restores Neurogenesis in the Aged Rat Brain. Mol. Psychiatry.

[30] Lommatzsch M., Zingler D., Schuhbaeck K., Schloetcke K., Zingler C., Schuff-Werner P., Virchow J.C. (2005). The Impact of Age, Weight and Gender on BDNF Levels in Human Platelets and Plasma. Neurobiol. Aging.

[31] Komulainen P., Pedersen M., Hänninen T., Bruunsgaard H., Lakka T.A., Kivipelto M., Hassinen M., Rauramaa T.H., Pedersen B.K., Rauramaa R. (2008). BDNF Is a Novel Marker of Cognitive Function in Ageing Women: The DR’s EXTRA Study. Neurobiol. Learn. Mem..

[32] Zhang Y., Gu F., Chen J., Dong W. (2010). Chronic Antidepressant Administration Alleviates Frontal and Hippocampal BDNF Deficits in CUMS Rat. Brain Res..

[33] Zeng Y., Zhao D., Xie C.-W. (2010). Neurotrophins Enhance CaMKII Activity and Rescue Amyloid-β-Induced Deficits in Hippocampal Synaptic Plasticity. J. Alzheimer’s Dis..

[34] Arancibia S., Silhol M., Moulière F., Meffre J., Höllinger I., Maurice T., Tapia-Arancibia L. (2008). Protective Effect of BDNF against Beta-Amyloid Induced Neurotoxicity in Vitro and in Vivo in Rats. Neurobiol. Dis..

[35] Eide F.F., Vining E.R., Eide B.L., Zang K., Wang X.-Y., Reichardt L.F. (1996). Naturally Occurring Truncated TrkB Receptors Have Dominant Inhibitory Effects on Brain-Derived Neurotrophic Factor Signaling. J. Neurosci..

[36] Holsinger R.M.D., Schnarr J., Henry P., Castelo V.T., Fahnestock M. (2000). Quantitation of BDNF MRNA in Human Parietal Cortex by Competitive Reverse Transcription-Polymerase Chain Reaction: Decreased Levels in Alzheimer’s Disease. Mol. Brain Res..

[37] Ginsberg S.D., Che S., Wuu J., Counts S.E., Mufson E.J. (2006). Down Regulation of Trk but Not P75 NTR Gene Expression in Single Cholinergic Basal Forebrain Neurons Mark the Progression of Alzheimer’s Disease: Down Regulation of Trk. J. Neurochem..

[38] Peng S., Wuu J., Mufson E.J., Fahnestock M. (2005). Precursor Form of Brain-Derived Neurotrophic Factor and Mature Brain-Derived Neurotrophic Factor Are Decreased in the Pre-Clinical Stages of Alzheimer’s Disease: Decreased ProBDNF and BDNF in Alzheimer’s Disease. J. Neurochem..

[39] Ferrer I., Marín C., Rey M.J., Ribalta T., Goutan E., Blanco R., Tolosa E., Martí E. (1999). BDNF and Full-Length and Truncated TrkB Expression in Alzheimer Disease. Implications in Therapeutic Strategies. J. Neuropathol. Exp. Neurol..

[40] Connor B., Young D., Lawlor P., Gai W., Waldvogel H., Faull R.L.M., Dragunow M. (1996). Trk Receptor Alterations in Alzheimer’s Disease. Mol. Brain Res..

[41] Shlomi S., Toledano R., Nitzan K., Dror Shahaf S., Break E.P., Frenkel D., Doron R. (2022). Imbalance in Sirt1 Alternative Splicing in Response to Chronic Stress during the Adolescence Period in Female Mice. Int. J. Mol. Sci..

[42] Sarne Y. (2019). Beneficial and Deleterious Effects of Cannabinoids in the Brain: The Case of Ultra-Low Dose THC. Am. J. Drug Alcohol Abus..

[43] Quesseveur G., David D.J., Gaillard M.C., Pla P., Wu M.V., Nguyen H.T., Nicolas V., Auregan G., David I., Dranovsky A. (2013). BDNF Overexpression in Mouse Hippocampal Astrocytes Promotes Local Neurogenesis and Elicits Anxiolytic-like Activities. Transl. Psychiatry.

[44] Yang J., Siao C.-J., Nagappan G., Marinic T., Jing D., McGrath K., Chen Z.-Y., Mark W., Tessarollo L., Lee F.S. (2009). Neuronal Release of ProBDNF. Nat. Neurosci..

[45] Bramham C.R., Messaoudi E. (2005). BDNF Function in Adult Synaptic Plasticity: The Synaptic Consolidation Hypothesis. Prog. Neurobiol..

[46] Rossi C., Angelucci A., Costantin L., Braschi C., Mazzantini M., Babbini F., Fabbri M.E., Tessarollo L., Maffei L., Berardi N. (2006). Brain-Derived Neurotrophic Factor (BDNF) Is Required for the Enhancement of Hippocampal Neurogenesis Following Environmental Enrichment. Eur. J. Neurosci..

[47] Huang E.J., Reichardt L.F. (2001). Neurotrophins: Roles in Neuronal Development and Function. Annu. Rev. Neurosci..

[48] Cao T., Matyas J.J., Renn C.L., Faden A.I., Dorsey S.G., Wu J. (2020). Function and Mechanisms of Truncated BDNF Receptor TrkB.T1 in Neuropathic Pain. Cells.

[49] Fenner M.E., Achim C.L., Fenner B.M. (2014). Expression of Full-Length and Truncated TrkB in Human Striatum and Substantia Nigra Neurons: Implications for Parkinson’s Disease. J. Mol. Histol..

[50] Martinez-Cengotitabengoa M., MacDowell K.S., Alberich S., Diaz F.J., Garcia-Bueno B., Rodriguez-Jimenez R., Bioque M., Berrocoso E., Parellada M., Lobo A. (2016). BDNF and NGF Signalling in Early Phases of Psychosis: Relationship With Inflammation and Response to Antipsychotics After 1 Year. Schizophr. Bull..

[51] Salter M.W., Stevens B. (2017). Microglia Emerge as Central Players in Brain Disease. Nat. Med..

[52] Parkhurst C.N., Yang G., Ninan I., Savas J.N., Yates J.R., Lafaille J.J., Hempstead B.L., Littman D.R., Gan W.-B. (2013). Microglia Promote Learning-Dependent Synapse Formation through Brain-Derived Neurotrophic Factor. Cell.

[53] Stella N. (2010). Cannabinoid and Cannabinoid-like Receptors in Microglia, Astrocytes, and Astrocytomas. Glia.

[54] Tselnicker I., Keren O., Hefetz A., Pick C.G., Sarne Y. (2007). A Single Low Dose of Tetrahydrocannabinol Induces Long-Term Cognitive Deficits. Neurosci. Lett..

[55] Ozaita A., Aso E. (2017). The Cannabis Paradox: When Age Matters. Nat. Med..

[56] Calabrese E.J., Rubio-Casillas A. (2018). Biphasic Effects of THC in Memory and Cognition. Eur. J. Clin. Investig..

[57] Oakley H., Cole S.L., Logan S., Maus E., Shao P., Craft J., Guillozet-Bongaarts A., Ohno M., Disterhoft J., Van Eldik L. (2006). Intraneuronal Beta-Amyloid Aggregates, Neurodegeneration, and Neuron Loss in Transgenic Mice with Five Familial Alzheimer’s Disease Mutations: Potential Factors in Amyloid Plaque Formation. J. Neurosci..

[58] Chernyuk D.P., Bol’shakova A.V., Vlasova O.L., Bezprozvanny I.B. (2021). Possibilities and Prospects of TheBehavioral Test “Morris Water Maze”. J. Evol. Biochem. Physiol..

