# Supplementary material for: An Ultra-Low Dose of ∆9-Tetrahydrocannabinol Alleviates Alzheimer’s Disease-Related Cognitive Impairments and Modulates TrkB Receptor Expression in a 5XFAD Mouse Model
Source: Int J Mol Sci. 2022 Aug 21;23(16):9449. doi: 10.3390/ijms23169449 (PMC9408848; doi:10.3390/ijms23169449)
Supplement: Supplementary file 1 [file ijms-23-09449-s001.zip › ijms-1855472-supplementary.pdf]

Supplementary Material

1 Motor functions

Motor function of 6-month-old mice (experiment 1) was evaluated both in the open field and in the Morris water maze. There were no differences in motor performance between the groups. Motor functions were evaluated using the total movement in the open field test (Figure S1A) and velocity in the last day of the MWM test (Figure S1B).

<u>Figure S1</u>

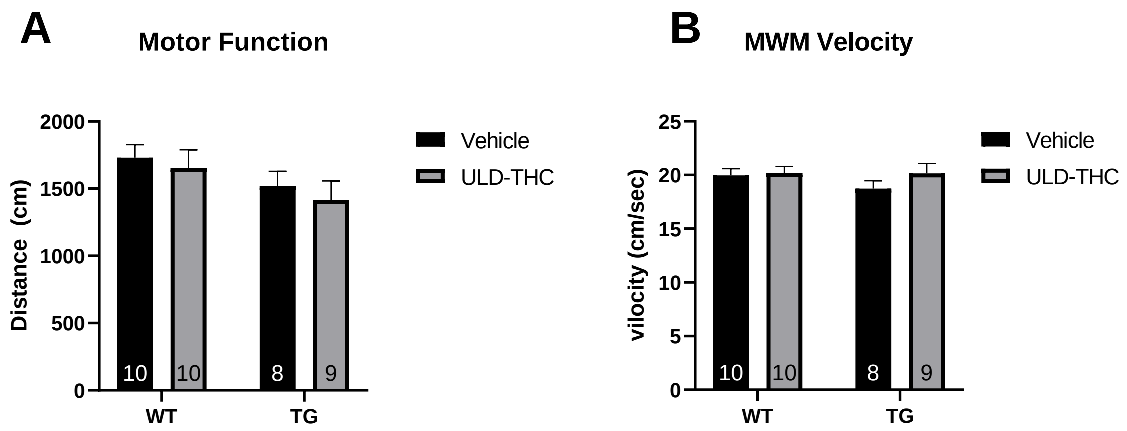

Figure S1 - Motor functions at 6 months. No difference was found in motor function, neither in the distance moved in the open field (A) or in the velocity on the last day of the MWM (B). Mean +/- SEM

Motor function of 12-month-old mice (experiment 2) was evaluated both in the open field and in the Morris water maze (Figure S2). No difference was found in motor function in the distance moved in the open field (Figure S1A). On the last day of the MWM (Figure S1A), two-way ANOVA yielded a significant difference between the groups ($F(1,30) = 4.819$, $p = 0.03$), with the only difference being between the WT and the 5xFAD group ($p = 0.03$).

Figure S2

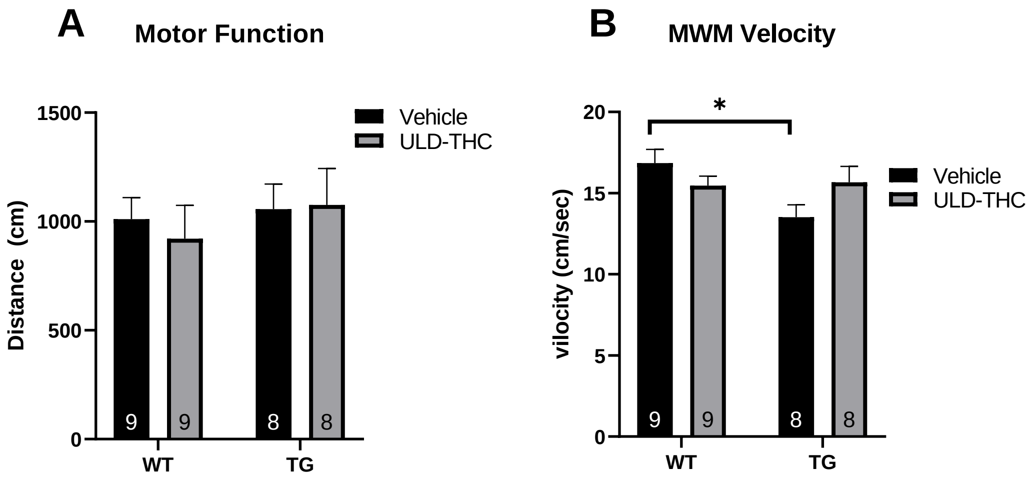

Figure S2 – Motor functions at 12 months old. No difference was found in motor function in the distance moved in the open field (A). On the last day of the MWM (B), there was a small but significant difference in swim velocity between the WT group and the 5xFAD group. Mean +/- SEM.* indicates $p<0.05$

2 Information on 12 months old males and females

The number of male and female mice in each group is detailed in Table S1.

Table S1 - The number of male and female mice in each group.

n	WT	WT treated	5xFAD	5xFAD treated
Females	4	4	4	3
Males	5	5	4	5

There were no significant differences in the performance between the genders.

Two-way ANOVA yielded no differences between the genders in the Y maze (Figure S3A). Likewise, in the MWM, repeated three-way ANOVA yielded no differences between the genders over all the testing days (Figure S3B).

Figure S3

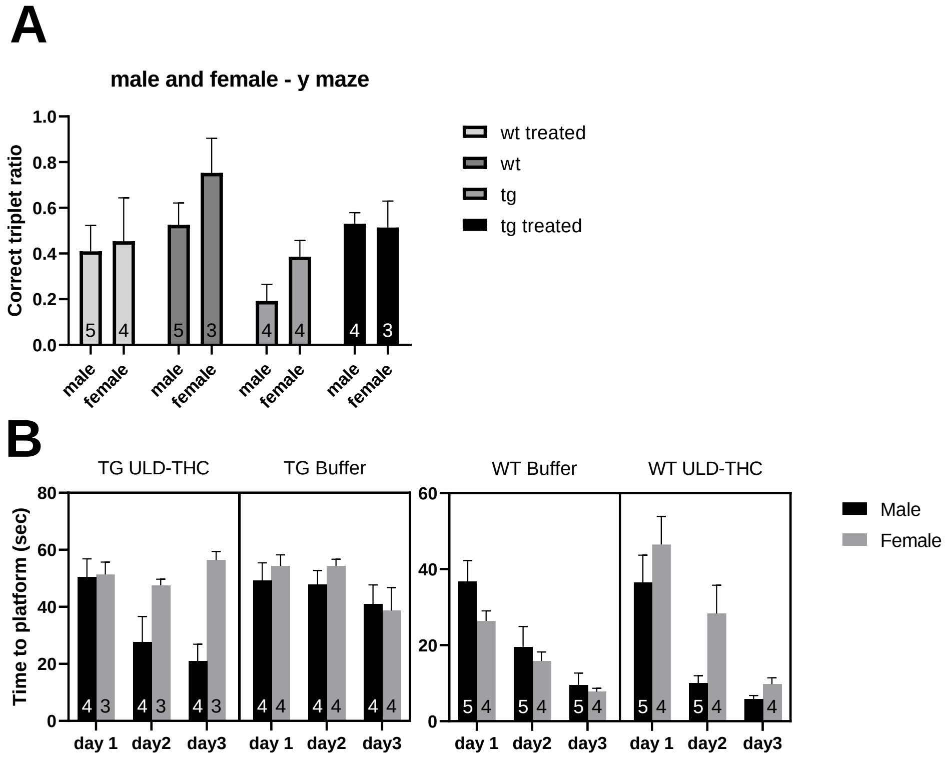

Figure S3 - Male and female performance at 12 months. No difference was found between the genders in the Y maze (A) or in the MWM (B). Mean +/- SEM.
